# Development and characterization of an oat TILLING-population and identification of mutations in lignin and β-glucan biosynthesis genes

**DOI:** 10.1186/1471-2229-10-86

**Published:** 2010-05-12

**Authors:** Aakash Chawade, Per Sikora, Marcus Bräutigam, Mikael Larsson, Vivekanand Vivekanand, Montedar All Nakash, Tingsu Chen, Olof Olsson

**Affiliations:** 1 Department of Cell and Molecular Biology, Göteborg University, S-40530 Göteborg, Sweden; 2 Department of Plant and Environmental Sciences, Göteborg University, S-40530, Göteborg, Sweden; 3 CropTailorAB, Erik Dahlbergsgatan 11A, SE41126 Göteborg, Sweden; 4 Department of Chemical and Biological Engineering, Chalmers University of Technology, S-41296, Göteborg, Sweden; 5 Current Address: Microbiology Research Institute, Guangxi Academy of Agricultural Sciences, Nanning, Guangxi 530007, China

## Abstract

**Background:**

Oat, *Avena sativa *is the sixth most important cereal in the world. Presently oat is mostly used as feed for animals. However, oat also has special properties that make it beneficial for human consumption and has seen a growing importance as a food crop in recent decades. Increased demand for novel oat products has also put pressure on oat breeders to produce new oat varieties with specific properties such as increased or improved β-glucan-, antioxidant- and omega-3 fatty acid levels, as well as modified starch and protein content. To facilitate this development we have produced a TILLING (Targeting Induced Local Lesions IN Genomes) population of the spring oat cultivar SW Belinda.

**Results:**

Here a population of 2600 mutagenised M2 lines, producing 2550 M3 seed lots were obtained. The M2 population was initially evaluated by visual inspection and a number of different phenotypes were seen ranging from dwarfs to giants, early flowering to late flowering, leaf morphology and chlorosis. Phloroglucinol/HCl staining of M3 seeds, obtained from 1824 different M2 lines, revealed a number of potential lignin mutants. These were later confirmed by quantitative analysis. Genomic DNA was prepared from the M2 population and the mutation frequency was determined. The estimated mutation frequency was one mutation per 20 kb by RAPD-PCR fingerprinting, one mutation per 38 kb by MALDI-TOF analysis and one mutation per 22.4 kb by DNA sequencing. Thus, the overall mutation frequency in the population is estimated to be one mutation per 20-40 kb, depending on if the method used addressed the whole genome or specific genes. During the investigation, 6 different mutations in the phenylalanine ammonia-lyase (*AsPAL1*) gene and 10 different mutations in the cellulose synthase-like (*AsCslF6*) β-glucan biosynthesis gene were identified.

**Conclusion:**

The oat TILLING population produced in this work carries, on average, hundreds of mutations in every individual gene in the genome. It will therefore be an important resource in the development of oat with specific characters. The population (M5) will be available for academic research via Nordgen http://www.nordgen.org as soon as enough seeds are obtained.

[Genbank accession number for the cloned *AsPAL1 *is GQ373155 and GQ379900 for *AsCslF6*]

## Background

The genus *Avena *belongs to the grass family Poaceae [1]. It comprises about seventy species, although mainly *A. sativa*, *A. nuda *and *A. byzantina *are those most commonly cultivated on a commercial scale. *A. sativa *is an economically important crop and ranks sixth in world cereal production after wheat, maize, rice, barley and sorghum [2]. Oat is grown for grain and hay and is mostly used as a feed for cattle. In 2008-2009, oat was cultivated on 12.76 million hectares mostly in temperate areas in the USA, Canada and Europe, but also on significant areas in China, Brazil and Australia [3]. Oat is well adapted to a wide range of soil types and performs better than many other small grain cereals on both acidic and alkaline soils [4].

Oat seeds have good nutritional value with a high content of essential dietary minerals, unsaturated fatty acids, unique galacto-lipids and the highest levels of globular proteins amongst any cereal. They also have high levels of mixed (1→3), (1→4) β-D-glucans [5], which are beneficial for digestion and have cholesterol-lowering properties [6-8]. Moreover, oat contains compounds such as tocopherols, inositol phosphates and avenanthramides which possess antioxidative properties [9].

In the last few years, the awareness-increase of the health-beneficial properties of oats has lead to a heightened consumption in Great Britain and the Scandinavian countries [10]. Even though traditional oat breeding has been successful in continuously improving the beneficial properties of oat, further enhancement is possible. A winter oat may increase the average yield by more than 20% [11,12], a more easily digestible kernel containing a low lignin hull would increase the energy value of the feed by 15% [13,14], an oat rich in β-glucan fibres would further increase the health beneficial properties for humans [6-8] and a low mycotoxin oat would exclude oat products from the new threshold values for mycotoxin levels that will be introduced in the European Union during 2010 [15].

Application of molecular techniques in oat breeding would facilitate the development of more complex characteristics. Although marker assisted mapping programs are in progress [16,17], generation of mutated oat lines by transposons, T-DNA or RNA interference techniques is technically difficult and has not been attempted. This is partly due to the lack of an efficient transformation system and partly due to the large genome size with an estimated 1C genome weight of 13.23 pg, corresponding to about 13,000 Mbp [18]. The fact that cultivated oat is hexaploid [19] and the genome not sequenced complicates the matter even further. Therefore, although several oat ESTs are publicly available, many more will be required to cover the entire transcriptome [20].

One way to increase variation in the breeding process would be to use radiation or chemical mutagens such as EMS (ethyl methanesulfonate). EMS is a highly mutagenic substance that preferentially alkylates guanine bases leading to the DNA-polymerase favoring the placement of a thymine residue instead of a cytosine residue opposite to the O-6-ethyl guanine in the subsequent DNA-replication step. This results in a random point mutation wherein G-C base-pairs (bps) are switched to A-T pairs [21]. Mutations in coding regions can be silent, missense or nonsense and mutations outside coding regions, like promoter mutations resulting in up- or down-regulation of transcription, aberrant splicing of mRNA, altered mRNA stability or changes in protein translation may also occur.

In TILLING, high frequency mutagenesis is combined with molecular based, high precision selection methods. The power of TILLING was first demonstrated in *Arabidopsis thaliana *[22] and *Drosophila melanogaster *[23] and has later been successfully applied to a number of plant systems including *Arabidopsis *[24], barley [25,26], *Lotus japonicus *[27,28], wheat [29], maize [30], rice [31,32], pea [33], soybean [34] and naked oats [35]. If the mutation frequency is high enough, and the population size large enough, a mutated allele of most, if not all, genes will be present in the population. In combination with an efficient method to identify point mutations, for example the Li-COR method [22], it is possible to apply TILLING to genetically complicated crops as well. The power of the principle was elegantly confirmed by the identification of number of waxy gene mutants in TILLING populations of both tetraploid and hexaploid wheat where the corresponding waxy enzymes in some cases completely lost their activity [29]. Moreover, a plant carrying a triple mutant, which displayed a near null waxy phenotype was also developed [29]. A TILLING-population in a hexaploid oat with a reasonable mutation frequency should therefore, by analogy, be a very useful breeding tool. Although Li-COR based techniques are commonly used for screening for mutations, MALDI-TOF (Matrix-assisted laser desorption/ionization-time-of-flight mass spectrometer) based assays can also be used. In the MALDI-TOF, samples are ionised and then relocated to the mass analyser where they are separated according to their mass to charge ratio. The ions are then detected and analysed by means of specially developed software.

In this work, a TILLING population for hexaploid oat, consisting of 2550 different mutagenised seed lines was developed and the mutation frequency determined. To screen the population, a method based on MALDI-TOF MassCLEAVE protocol™ [36] was adapted for TILLING. We demonstrated the potential of the oat TILLING population by identifying several different mutations in the oat *AsPAL1 *and *AsCslF6 *genes - key genes in the lignin and β-glucan biosynthetic pathways, respectively. These genes were chosen because they encode traits of great interest to oat breeders. A down-regulation of the *PAL *will result in modified, more easily extractable lignins with increased digestibility [37,38], which will in-turn increase the feed value of the crop. Since β-glucans are becoming very important functional food ingredients, an oat variety with increased or modified β-glucan content in the seed will significantly increase the value of the crop. Oat β-glucans have a health claim both in the US and in the EU and a regular consumption of oat β-glucans has been linked to a number of health beneficial effects like lowering of blood cholesterol levels, a decrease of the general glycemic index of ingested food and some protection against colon cancer [39].

## Results

### Development of an oat TILLING-population

**Figure 1:**
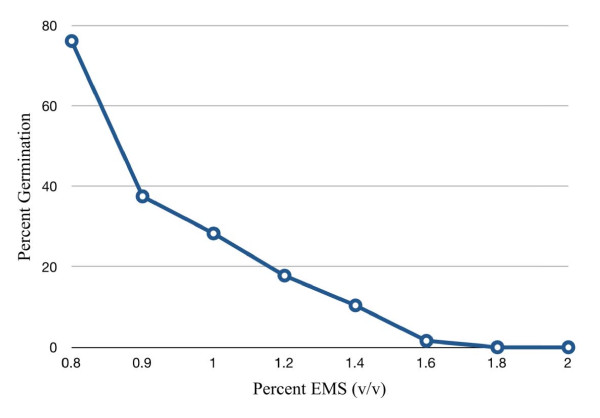
**Titration of EMS concentration**. Y-axis: % germinating seeds, X-axis: % EMS concentration

To determine the optimal survival rate for TILLING-applications, an EMS titration kill curve for oat seeds was established at different EMS concentrations (Figure 1). In total 8 different EMS concentrations were used and ~200 seeds were treated at each concentration. An EMS concentration of 0.9% (v/v) was finally chosen for large-scale mutagenesis, giving a predicted survival rate of approximately 37% (Figure 1). Approximately 9000 seeds were mutagenised, germinated and the surviving M1 plants were allowed to set seeds. Of the 2880 mutants that germinated, 2800 mutants produced filled panicles, while the rest died, did not flower or produced empty panicles.

**Figure 2:**
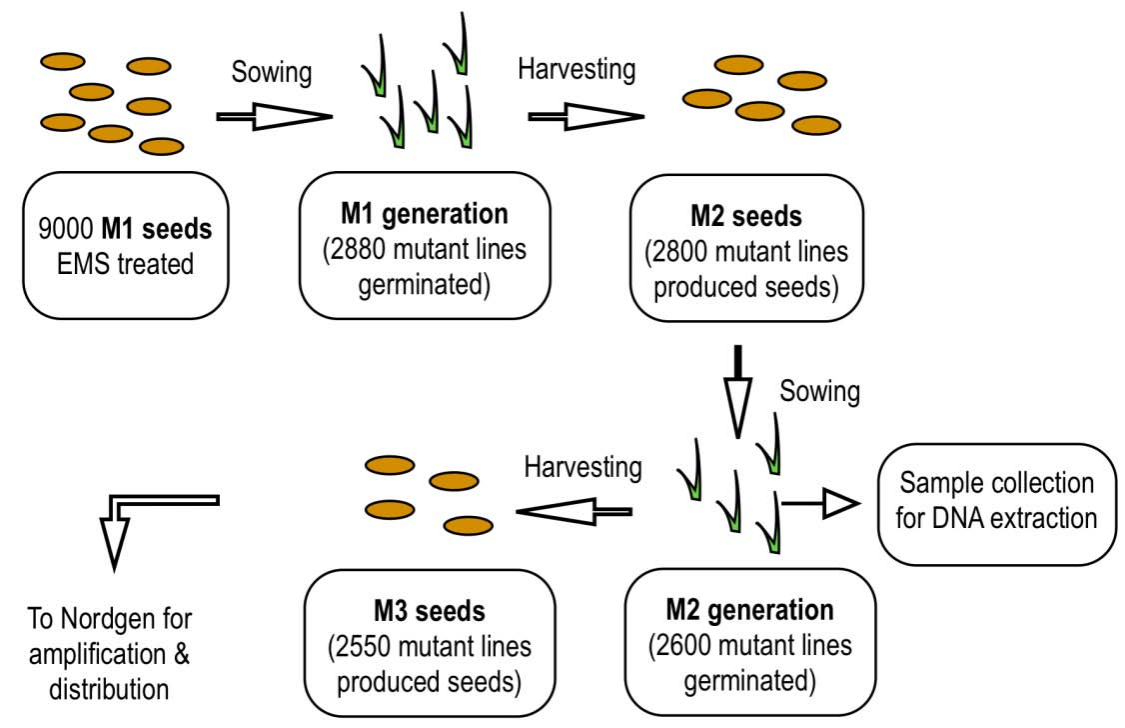
**Schematics of TILLING population development**. Figure shows the schematics for the step-wise preparation of the TILLING library. Upon EMS treatment, M1 seeds were sown in soil and M1 generation plants allowed to self-pollinate. M2 seeds from individual line were collected separately and one seed per line sown in soil. Once germinated, leaf samples were collected for DNA preparation and the plants allowed to set seeds. M3 seeds were then collected and stored. Preparations are underway to setup the seed database at Nordgen.

**Figure 3:**
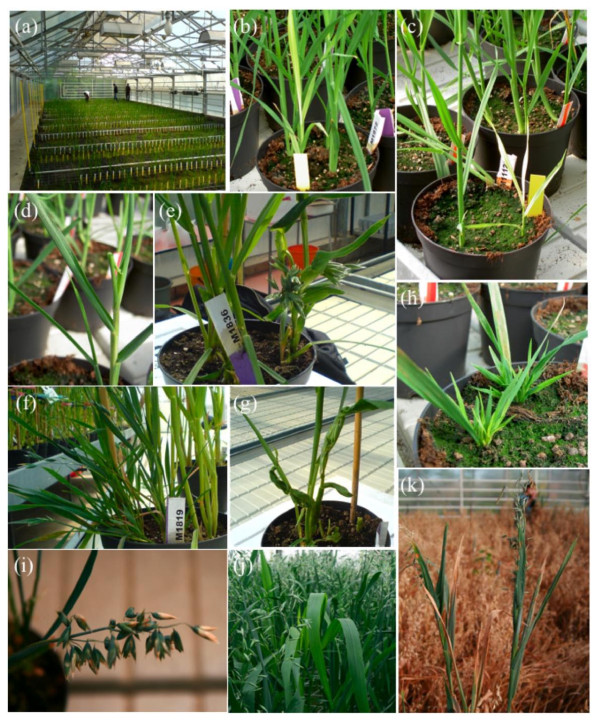
**Examples of phenotypes seen in the M2 segregating oat Tilling population**. (a) M2 population growing up (about 50% of the total population shown). (b) and (c) Chlorotic mutation. (d) - (g) Morphological mutations. (h), (i) Dwarf. (j), (k) Broad leaved and tall

M2 seeds were harvested from each of the M1 lines and used to plant out the M2 population. Out of 2800 seeds planted, 2600 germinated (Figure 2). The entire M2 life cycle was then followed and phenotypic abnormalities on individual plants in the segregating population were recorded. Normally, phenotypic changes are extremely rare in a population of non-mutated Belinda that has been inbred for generations (personal communication with oat breeders). However, in the TILLING-population chlorosis (2.3%), semi-dwarfness (1.3%), giants (0.6%), dwarf and high tiller (0.6%), late/early flowering and late senescence were frequently observed (Figure 3). In total, ca 5% of the mutagenised Belinda population had visually detectable phenotypic alterations.

Genomic DNA was extracted from two-week-old leaves from each of the 2600 different lines in the M2 population by the cetyltrimethylammonium bromide (CTAB) method [40]. After flowering and seed production an average of 100 seeds (varying from 5 to 150) were harvested from each of the 2550 fertile lines. These seeds make up the M3 TILLING-population (Figure 2).

### Biochemical analysis of lignin content

**Figure 4:**
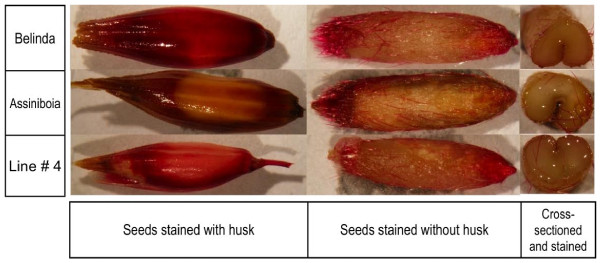
**Phloroglucinol staining of a mutant with low-lignin content**. Picture shows phloroglucinol stained seeds from Belinda, Assiniboia and a mutant with low lignin content. Seeds were either stained with husk or first de-husked and then stained. Alternatively, seeds were de-husked, cross-sectioned and then stained.

The Wiesner phloroglucinol-HC1 assay [41] was used to screen for mutants with differential lignin deposition in the seed coat. M3 seeds from each line of approximately 70% of the population (1824 independent, random M2 mutagenised lines) were stained (see methods). Typically, whole seeds, whole de-husked seeds and de-husked cross-sectioned seeds were tested. Non-mutated Belinda was used as a positive high lignin control and the Canadian variety A. C. Assiniboia (AAFC, Canada) as a low lignin control. From these, 17 mutant lines were identified that showed lower stain intensity in the husk compared to Belinda. A representative result is shown in Figure 4.

### Lignin quantification

**Figure 5:**
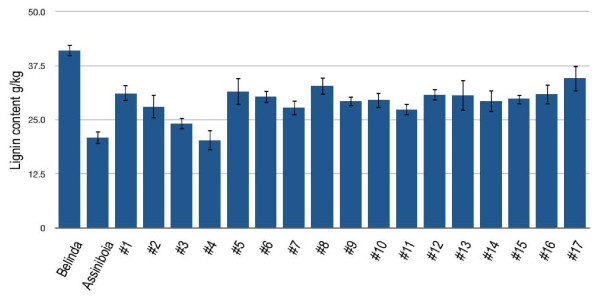
**Lignin quantification by acetyl bromide method**. Chart shows the lignin quantification done using acetyl bromide method in different lines. Belinda and Assiniboia used as controls where as rest of the 18 lines are from the mutant population. Ten seeds per line were tested. Error bars are the standard errors.

Although phloroglucinol-HCl assay is a rapid method, it is not quantitative. Therefore, the lignin content in the mutant candidates was measured with the acetyl bromide method. This method is relatively simple and appropriate for small sample sizes. The method allows complete dissolution of lignin and hence provides precise absorbance values for total lignin content with little interference from non-lignin products [42]. For each line, 10 individual seeds were weighed and measured. The lignin content in Belinda was 41.04 g/kg and 20.84 g/kg in Assiniboia, a Canadian low lignin variety. This is in good agreement with previous lignin tests on Assiniboia [43]. In the identified mutant lines, lignin contents were in the range of 20-34 g/kg. Line #4 was the lowest with 20.31 g/kg, which is in the same range as in Assiniboia (Figure 5).

### Estimation of the mutation frequency by RAPD-PCR

Even though the number of visual phenotypes in a population indirectly reflects the number of mutations in the genome, it is not feasible to estimate the mutation frequency in this way due to gene redundancy, silent mutations and the fact that oat is hexaploid. Therefore, to better approximate the mutation frequency a RAPD-PCR (Random Amplification of Polymorphic DNA) method was exploited. Briefly, using short random 10 bp primers, chromosomal DNA from non-mutated Belinda was amplified. After standardizing PCR reactions with Belinda wt DNA as a template, 16 different bands were routinely obtained with the particular primer combination used. When analyzing DNA from 252 independent lines using the same PCR conditions, 4 mutants were found where the band pattern differed from non-mutagenised Belinda (data not shown). A gain or loss of a band will occur if the particular chromosomal sequence to which the random primer binds, has at least one mutated bp. Assuming a target size of 20 bp (2 primers), 16 bands covers a target of 320 bp. One mutation per 63 lines therefore indicates an average mutation frequency in each line of approximately one mutation per 20 kb.

### Cloning of PAL and CslF genes

**Figure 6:**
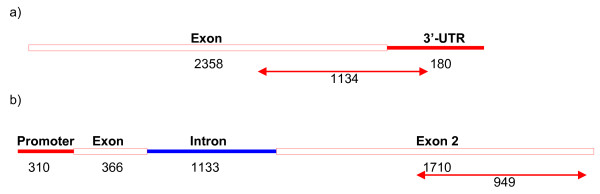
**Schematic map of the genes used for screening**. Red line, 5' upstream and 3' downstream sequence; red box, exon sequence; blue line, intron sequence. The sizes of the different regions are indicated. Double arrows indicate the PCR fragments amplified for mutation screening. The sizes of the fragments are indicated below the arrows. a) *AsCsFl6*. b) *AsPAL1*

A genomic *AsPAL1 *sequence of 3209 bp including one 1133 bp long intron (Figure 6a) and a 2358 bp long partial *AsCslF6 *mRNA was obtained (Figure 6b). The sequences of both genes have been submitted to Genbank, accession number for *AsPAL1 *is GQ373155 and GQ379900 for *AsCslF6*.

### Mutant discovery by MALDI-TOF analysis

**Figure 7:**
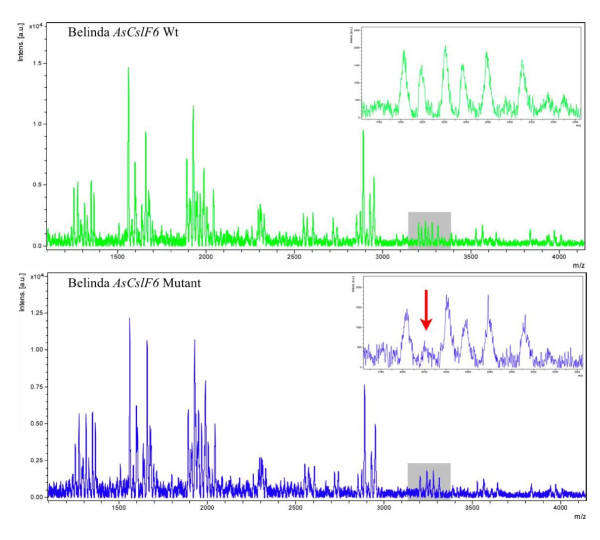
**Identification of single nucleotide differences in the AsCslF6 gene by MALDI-TOF**. Amplified PCR fragments were transcribed, cleaved at every Uracil-residue and separated according to size. X- axis indicate the MW of each fragment in Daltons (Da) and the Y-axis denotes intensity in Arbitrary Units (a.u.). The disappearance of an expected peak (red arrow) indicates the appearance of a new cleavage site in the fragment, i.e the introduction of a new Uracil residue. Grey box indicates magnified region.

Primers were designed and amplification products separated on agarose gels to confirm the expected product and to rule out unspecific products. Fragments with the expected size from several independent reactions were then sequenced to confirm that only a single sequence had been amplified. In the final PCR reactions, 949 bp from *AsCsFl6 *and 705 bp from the *AsPAL1 *gene were amplified (Figure 7) using DNA from the M2 population. During degradation with the adapted MassCLEAVE™ method before the MALDI-TOF fragment separation, 316 bp from *AsCslF6 *and 45 bp from *AsPAL1 *were in practice lost as some of the generated fragments became too short or too long to resolve satisfactorily in the MS. In total, 300 lines were analysed for mutations in the *AsCslF6 *gene and 350 lines for mutations in the *AsPAL1 *gene. This covered 190 kb of the *AsCslF6 *sequences and 231 kb of *AsPAL1 *sequences. The screenings yielded 5 *AsCslF6 *mutations and 6 *AsPAL1 *mutations, indicating a mutation frequency of one mutation in 38 kb and one mutation in 38.5 kb, respectively. The entire gene fragments from all identified mutants were DNA sequenced and the mutants confirmed.

### Confirmation of MALDI-TOF screening by DNA sequencing

To estimate the predictive abilities of the MALDI-TOF screening method, using the *AsPAL1 *gene, 160 of the lines that were screened with MALDI-TOF were subjected to DNA sequencing, adding to 113 kb. This not only confirmed the four *AsPAL1 *mutations that were previously identified but also showed that there were no other mutations that went undetected by the MALDI-TOF screening. Therefore it seems like the MALDI-TOF assay is sensitive enough to detect most mutations in a given sequence and that the background noise is low.

### Identification of mutations directly by DNA sequencing

Since the mutation frequency seemed to be high, we attempted to identify mutations directly by DNA sequencing. Using the *AsCslF6 *specific gene primers we amplified and sequenced the 949 bp fragment from 126 different lines, covering a total of 112 kb. This resulted in the identification of 5 additional *AsCslF6 *mutations, corresponding to one mutation per 22.4 kb.

### Characterisation of identified mutations

In the *AsPAL1 *sequence, four C to T and two G to A transitions were found. Three of these mutations were located in the wobbling base and therefore silent, but 3 led to an amino acid change. These mutations were evaluated by SIFT (Sorting intolerant from tolerant) [44] and PSSM (Position specific scoring matrix) [45] scores. Of the ten mutations found in *AsCslF6*, five created silent mutations and five corresponded to amino acid changes. Of these, four were G to A transitions and one an A to T transversion. The obtained PSSM scores of these mutations were not significant and thus it's unlikely that the identified mutations may cause any phenotypic changes. For further details see Table 1.

**Table 1:** Identified mutations in the AsPAL1 and AsCslF6 genes.

*Gene	Type	Amino acid	Position	PSSM Shift	SIFT	Method
*AsPAL1*	C to T	Thr to Ile	487	-6	Not Tolerated	MALDI-TOF and sequencing
*AsPAL1*	C to T	Thr to Met	532	0	Not Tolerated	MALDI-TOF and sequencing
*AsPAL1*	C to T	Pro to Ser	537	-	Tolerated	MALDI-TOF and sequencing
*AsPAL1*	C to T	Silent		-	-	MALDI-TOF and sequencing
*AsPAL1*	G to A	Silent		-	-	MALDI-TOF
*AsPAL1*	G to A	Silent		-	-	MALDI-TOF
*AsCslF6*	G to A	Val to Met		0	Tolerated	Sequencing
*AsCslF6*	G to A	Gly to Arg		-3	Tolerated	Sequencing
*AsCslF6*	C to T	Silent		-	-	Sequencing
*AsCslF6*	G to A	Silent		-	-	Sequencing
*AsCslF6*	G to A	Silent		-	-	Sequencing
*AsCslF6*	C to T	Silent		-	-	MALDI-TOF
*AsCslF6*	A to T	Met to Lys		-3	Tolerated	MALDI-TOF
*AsCslF6*	G to A	Silent		-	-	MALDI-TOF
*AsCslF6*	G to A	Gly to Asp		-2	Tolerated	MALDI-TOF
*AsCslF6*	G to A	Arg to His		-1	Tolerated	MALDI-TOF

*Gene: The genes screened for; Type: detected mutation; Amino acid: the predicted amino acid change in the protein; Position: Position of the changed amino acid in the protein; PSSM and SIFT: significance of change in the amino acids; Method: "MALDI-TOF and sequencing" indicates that the same mutation was identified by both MALDI-TOF and DNA sequencing. Other mutants were either identified by MALDI-TOF or by DNA sequencing, as indicated.

## Discussion

TILLING is a good alternative to more direct DNA modifying techniques since seed mutagenesis is easy to apply and relatively independent of genome size and organization [46]. To determine the feasibility of developing an oat TILLING population initial experiments were performed to find an optimal balance between mutation frequency and lethality. Since the oat genome size is very large, it is crucial that the mutation frequency in the final TILLING-population is high. Our aim was to produce a population where mutations in all genes can be found with a high redundancy without having to produce a very large population size, which requires more advanced logistics in handling and maintenance. Screening large populations also becomes laborious, expensive and technically more demanding. On the other hand, a too high mutation frequency will increase the frequency of deleterious mutations, which if too numerous will kill the plant. After testing different EMS concentrations we settled for 0.9% (v/v) or 86.98 mM, which gave a germination rate of 37%. This EMS concentration is in the range of what has been used for wheat (0.75%, 1.0% or 1.2% v/v) [29] and maize (1% v/v) [30], whereas for barley three different concentrations were used (10, 20, 30 mM corresponding to 0.1%, 0.2%, 0.3% v/v) [25]. The concentration used for rice was much higher (1.5% v/v) [32].

In *Avena *four genomes, denoted A, B, C and D, have been identified. In nature, 5 different combinations of these genomes, namely AA, CC, AABB, AACC and AACCDD can be found [47]. Belinda, like other commonly cultivated *A. sativa *species, is a natural allo-hexaploid that contains three genomes AA, CC and DD. Such a redundant genomic organization could be a potential complication when identifying specific phenotypes from the TILLING-population. The presence of a specific allele on all three genomes would mean that even if one is mutated other alleles could compensate for the lost function. However, as has been shown in hexaploid wheat, the three genomes often contribute differently to the expression of certain alleles, which also can also vary even in different tissues of the plant [48]. When inspecting the oat M2 segregating TILLING-population visible phenotypes were indeed observed in about 5% of the plants (Figure 2). This is surprisingly high considering that in wheat visible phenotypes were seen in only 0.5% of the cases [29]. To extend the resolution of the phenotypic observations we therefore decided to test a specific biochemical pathway.

We stained individual M3 seeds from 1824 randomly chosen individual lines with phloroglucinol-HCI (Wiesner test) [41]. This test is generally considered to be indicative of aldehyde end groups present in, for example cinnamyl aldehydes and is thus indicative of lignin content and/or structure [49]. The Wiesner test revealed differences in the staining pattern in approximately 1% of the screened seeds. Again, this is a surprisingly high frequency, which corroborated the phenotypic observations. To verify this, total lignin in seeds from the mutant lines were quantified with the acetyl bromide method. In this assay, bromine replacement of α-carbon-OH groups produce a brown-red colored complex, which is dissolved in acetic acid. The intensity of the color is proportional to the amount of lignin [50]. The assay confirmed all 17 of the earlier identified mutants (Figure 5). Since it is not likely that independent mutations have simultaneously occurred in the same lignin gene in all of the three oat genomes, this indicates that at least some mutations are dominant in a way that is normally seen in a diploid plant. More studies are needed to determine if oat is different from wheat in this respect.

To test the mutation frequency more directly on the DNA level, two different approaches were used - RAPD and MALDI-TOF. One was more generally aimed at the whole genome, while the other was directed at specific genes.

By optimising a RAPD-PCR based method for oat genomic DNA the average mutation frequency for the whole genome was calculated. An advantage with this method is that it is relatively fast and simple. When analysing DNA samples from 252 different lines, 4 abnormal electrophoresis patterns were found, corresponding to a mutation frequency of approximately 1 per 20 kb (data not shown) assuming that only a single bp is sufficient to prevent primer binding. However, this might not always be the case and therefore the calculated mutation frequency will rather underestimate than overestimate the true frequency.

To compare the genome wide mutation frequency to the frequency in specific genes, two different genes, each encoding characteristics important for oat breeders, were chosen as models. The first gene, *AsPAL1*, encodes a protein that catalyses the first step of the phenyl-propanoid pathway [51]. The second gene, *AsCslF6*, encodes an enzyme involved in the biosynthesis of mixed link 1-3, 1-4 β-D-glucans (β-glucans). β-glucans are water-soluble fibres common in the cell wall of the *Poaceae *family and are synthesised by one or several members of a very large cellulose synthase superfamily, from which the *CslC*, *CslH *and *CslF *genes seem to be most relevant [52,53]. These two genes were screened for mutations by MALDI-TOF.

By adapting a MALDI-TOF based SNP screening method to oat TILLING, and by direct DNA sequencing, mutations for the two target genes were identified. In total 533 kb were covered and 16 mutations were found (Table 1), giving an average mutation frequency of 1 per 33.3 kb. This discrepancy from the RAPD-PCR estimation could reflect the fact that, in contrast to the genome wide estimate where local differences are evened out, factors such as chromosomal location, degree of closed or open chromatin, gene redundancy level etc., can influence the result when focusing on more specific regions. Additional investigations are therefore needed to explain more precisely why the mutation frequency in the chosen genes appears to be lower than the general frequency.

Overall, the mutation frequency in the oat TILLING-population thus appears to be in the same range as previously found in hexaploid wheat (1 mutation/24 kb) [29]. This is much higher than what was described in barley (1 mutation/1000 kb) [25], maize (1 mutation/500 kb) [30], rice (1 mutation/530 kb) [32] and other dicots [28,33,54]. The specific reasons for these differences are not yet known, but presumably larger polyploid genomes like oat and wheat can tolerate more mutations before lethality occurs.

Successful TILLING not only depends on the population size and the mutation frequency, the method used to identify specific mutations in the population is equally important. This is especially a concern with a large, hexaploid genome like oat. Up to now, all, published high-throughput TILLING procedures have utilized a CEL I based mismatch-cleavage enzyme on heteroduplex formations followed by a detection of end-labeled cleavage products on electrophoretic gels [30,54,55]. On the other hand MALDI-TOF based methods to identify SNP-mutations also have an inherent capacity to combine straightforward sample preparation with high sensitivity and high-throughput and are well documented, especially in clinical applications [36,56,57]. However, to our knowledge MALDI-TOF methods have not been systematically exploited in TILLING. One drawback with current MALDI-methods is their reliance on specialised equipment and software for identifying the mutations. Another potential problem for large scale screening programs is the high costs of the chemicals needed. On the other hand, MS based methods are sensitive, robust and reliable, which minimise redundant experiments and reduce the cost. They also allow the identification of homozygous mutations. In addition, using several alternative cleavage patterns from the same sample, mutations can be pinpointed down to the single bp level, potentially eliminating the need for confirmative DNA sequencing.

Thus, the MALDI-TOF method used here indeed detected mutations with an, up until now, 100% precision. The final percentage is likely to decrease somewhat as sample size increases. In any case, MALDI-TOF will be good complement or possibly an alternative to CEL I/Li-COR-screening provided that the per-sample cost could be brought down to an acceptable level and throughput increased. Without automation, MALDI-screening is still a low throughput method only allowing one person to screen approximately 288 samples per week, assuming 8-hour workdays. We are currently in the process of optimising and automating the protocol for large-scale screening.

A comprehensive analysis of the spectrum of mutations generated by EMS in *Arabidopsis *was published in 2003 [24]. It was shown that about 50% of all mutations were missense mutations that altered an amino acid, while approximately 5% of the mutations were nonsense resulting in premature termination of the target protein. Furthermore, in *Arabidopsis*, maize and wheat the majority of all mutations were the expected GC to AT transitions [29,30,54]. In rice on the other hand, this distribution was somewhat different and only 70% of recorded mutations were GC to AT [32]. In oat, we have so far identified 16 mutations, out of which 15 were GC to AT transitions (Table 1). Thus, oat seems to be similar to wheat in this respect. Most mutations were missense and silent. So far no nonsense mutation have been found (Table 1). We did not find any examples of small deletions or other rearrangements either, which indicates that the EMS mutagenesis worked as expected.

## Conclusions

In conclusion, a TILLING population of 2550 mutant lines containing a high frequency of mutations is presented. In combination with a high precision and efficient screening method, it is realistic to assume that productive mutations in all alleles in any gene of interest will eventually be identified. Since the genetic basis of a particular mutation then will be known, by repeated back crosses (introgression) especially productive mutations can be introduced to any chosen oat variety by marker assisted selection while the majority of the other mutations will be eliminated in the same process. Useful mutations in different alleles can also be crossed together. The oat tilling population will be deposited in NordGen http://www.nordgen.org as M5 seeds and will be available for academic purposes as soon as the population has been propagated. Additionally, a mutation-detection and screening service will be available to the scientific community for academic research through the company CropTailor AB http://www.croptailor.com.

## Methods

### Plant cultivation

Oat, *Avena sativa *v. Belinda a Swedish spring variety was obtained from the SW-collection (Svalöf Weibull AB, Landskrona, Sweden). Plants were grown in a greenhouse under halogen lamps, giving a photon flux density of 240 μmol/m2/sec with a photo-period of 18 h. Day and night temperatures were 25°C and 16°C respectively. Plants were grown in five-liter pots or directly on the ground in standard soil for oat growth.

### EMS mutagenesis

Fifty lots of ca 200 seeds were transferred to a 50 ml falcon tube and 30 ml of washing solution (0.01% v/v Tween 20 in water) was added to each tube. The tubes were gently shaken for 1 hr at 150 rpm at RT. The seeds were washed 2 times with water after which 10 ml 0.9% (v/v) or 86.98 mM EMS in water (M0880 Sigma Aldrich, Inc) was added to the tubes followed by shaking at 150 rpm for 16 hours at RT. The EMS solution was then discarded; seeds were washed three times with water and were vigorously shaken in water for 2 hrs. The water was discarded and the seeds were again washed 3 more times with water. The seeds rapidly loose their germination ability at this point and have to be immediately sown. This was done by carefully picking individual seeds with forceps and placing them in a small germination pot with standard soil for oat growth. Seeds were allowed to germinate and the number of germinating seeds was scored.

### Biochemical analysis

From the M3 generation two seeds (with husk) each from 1824 independent mutants were picked and stained with phloroglucinol-HCl reagent (1% phloroglucinol in 20% HCl) for 30 minutes as per the protocol [41,58] in 96 well plates. Seeds that stain different as compared to non-mutated Belinda were re-confirmed by staining several more seeds. For analyzing lignin deposition in the husk, seeds were stained without de-husking; for the seed coats, seeds were first de-husked and then stained, for cross-sections, seeds were de-husked, cross-sectioned and then stained. Upon staining, seeds were observed under a stereomicroscope (Olympus SZX-ILLB200).

### Lignin Quantification by Acetyl bromide method

Lignin was determined by acetyl bromide procedure as described [59] with few modifications. Briefly, from each line, ten seeds (hull+groat) were individually weighed and transferred to separate glass test tubes (16 × 150 mm) fitted with PTFE-coated silicone screw cap. Perchloric acid (70%, 0.08 ml) was then added followed by addition of 2 ml of acetyl bromide-glacial acetic acid (1:3 v/v) and incubated at 70°C for 15-20 min. The tubes were shaken gently every 5 minutes for complete dissolution. Contents were then transferred to 100 ml volumetric flasks containing NaOH (2 M, 5 ml) and acetic acid (12 ml). The final volume was made up-to 50 ml with acetic acid. Blank was run in conjugation with the samples without any seed. The absorbance was measured at 280 nm and lignin content was determined as described in Morrison et al. [60]. Means were measured from the ten seed samples and standard error calculated.

### Estimation of mutation frequency by RAPD analysis

Twelve random 10-mer primers, arbitrarily named Random 1, Random 2, etc, were obtained from MWG-Biotech in Ebersberg, Germany http://www.mwg-biotech.com. Initial RAPD-PCRs were performed on DNA from non-mutagenised Belinda in order to define primers that reproducibly gave around 20 bands in a range of DNA concentrations between 10 and 200 ng. In addition, effects of different concentrations of dNTP, Platinum Taq-polymerase (Invitrogen), MgCl_2_, different annealing temperature and number of cycles in the PCR-reaction on band pattern were tested. Random 6 was selected for further screening on genetic material isolated from the mutant library. The final protocol used was as follows: Random primer 6 (5'-AGCCAGCGAA-3', 40 mM), 10 ng DNA in a PCR buffer of (final concentration) 2.5 mM MgCl_2_, 0.8 mM dNTP, 2 μl Platinum Taq polymerase). An initial denaturation at 94°C for 2 minutes was followed by 45 cycles at 94°C for 30 seconds, 44°C for 1 minute, 72°C for 2 minutes and a final extension of 72°C for 5 minutes. Samples were held on ice until used. All reactions were performed in an Eppendorf Mastercycler Gradient. PCR-products were separated on a 2% agarose gel using Electrophoresis grade agarose dissolved in Tris-borate buffer at 4°C for 3 hrs at 6 V/cm and then stained using 3× GelRed solution (Bionuclear Scandinavia) for ca 45 minutes and photographed.

### *PAL *and *CslF *cloning

The partial sequence of the *PAL *was cloned from the homologue in *Zea mays *LOC542258 using the primers; 5'CCACGCGTCCGCTTC3', 5'CCACGAACACCTTGTCACAC3'. To elongate the obtained sequences Genome Walker™ (Clonetech Laboratories, Inc., CA, USA) kit was used. Using this kit, gDNA library was made as per the protocol with the restriction enzyme DRA I. The following nested primers were used to walk upstream; 5'GGGTCCTTCTCGTTGATGTGCC3', 5'TCGTTGATGTGCCCGTTGCC3' and primers used to walk downstream were; 5'TGGGGATCAGCCAGGGCAAG3', 5'CAAGCCGCTGCCCATCAACA3'. The PCR amplification was done in a 25 μl volume containing 5 ng of DNA from genome walker, 0.75 μl MgSO_4_, 2.5 μl HiFi taq buffer, 0.5 μl dNTP and 0.2 μl HiFi taq. PCR was conducted using the Eppendorf Mastercycler Gradient as follows: heat denaturation at 94°C for 2 min, followed by 30 cycles of PCR (94°C for 30 s, annealing temperature of 72°C for 30 s, extension temperature of 72°C for 4.5 min). PCR product was cloned using the TOPO-TA kit for sequencing (Invitrogen) and sent to MWG for sequencing. A candidate EST for a *CslF *gene was identified in the oat EST library [20]http://www.agod.org using tBLASTX. The *CslF*-gene was subsequently cloned and sequenced using the GeneRacer system (Invitrogen) to obtain the partial mRNA sequence. Samples were sent to MWG Biotech (Ebersberg, Germany) for sequencing and the two resulting fragments were assembled into a single sequence using sequence overlap. The translated sequence was compared to known *CslF *genes in rice, wheat and barley using tBLASTx for confirmation.

### PCR amplification for DNA sequencing and mutant analysis

Primers for *AsPAL1 *were; 5'CAGTAATACGACTCACTATAGGGAGAAGGCTCGCACCCGTCCCCTTAC3' and 5'TCCACGAACACCTTGTCACAC3'. The PCR amplification was done in a 25 μl volume containing 5 ng of genomic DNA, 0.75 μl MgSO_4_, 2.5 μl HiFi taq buffer, 0.5 μl dNTP and 0.2 μl HiFi taq. PCR was conducted using a Bio-Rad C1000 cycler as follows: heat denaturation at 94°C for 2 min, followed by 30 cycles of PCR (94°C for 30 s, annealing temperature of 65.7°C for 30 s, extension temperature of 68°C for 1 min). This resulted in a final fragment of 705 bp

The *CslF6 *gene was amplified using primers; 5'CGCACCCGTCCCCTTACG3' and 5'ACGACTGGCGTCTTTCCG3' as above but with an annealing temperature of 60.6°C, an extension time of 90 s and 35 cycles. One of the primers was placed in the 3' UTR of the gene to decrease the chances of amplifying multiple genomes. This resulted in a final fragment of 949 bp PCR amplified fragments were sent to Eurofins-MWG (Ebersberg, Germany) for sequencing. Chromatograms were assembled using the Sequencher application (Gene Codes Corporation) and visually inspected for unique base changes. After analysis, potential mutants were re-sequenced to confirm the existence of a mutated base.

### MALDI-TOF Analysis

To screen for mutations the MassCleave™ a modified protocol from the one originally released by SEQUENOM http://www.sequenom.com was used. More specifically, samples were amplified in a standard PCR-reaction using the previously mentioned primers with either the forward or reverse primer fused at the 5' end to a T7 binding domain: 5'CAGTAATACGACTCACTATAGGGAGAAGGCT3'. For *AsCslF6 *the PCR amplification was done in a 25 μl volume containing 10 ng of sample DNA, 0.75 μl MgCl_2_, 2.5 μl 10× taq buffer, 0.5 μl dNTP and 1 u Platinum Taq Polymerase (Invitrogen) using primers 5'CAGTAATACGACTCACTATAGGGAGAAGGCTCGCACCCGTCCCCTTACG3', 5'ACGACTGGCGTCTTTCCG3' for the forward reaction and 5'CGCACCCGTCCCCTTACG3', 5'CAGTAATACGACTCACTATAGGGAGAAGGCTACGACTGGCGTCTTTCCG3' for the reverse reaction. The PCR was conducted using a thermal cycler (Eppendorf Master Cycler Gradient) as follows: heat denaturation at 94°C for 2 min, followed by 45 cycles of PCR (94°C for 30 s, annealing temperature of 60.6°C for 30 s, extension temperature of 72°C for 1.3 min). For *AsPAL1 *the same protocol was used as described under sequencing and the primers 5'CAGTAATACGACTCACTATAGGGAGAAGGCTCTCCGAGCTCCAGT3', 5'CCACGAACACCTTGTCACAC3' for the forward reaction and 5'GAGAAGGACCCGCTCAACTG3', 5'CAGTAATACGACTCACTATAGGGAGAAGGCTAGAAGGCTCCACGAACACCTT3' for the reverse reaction. 5 ul of sample was treated with 1 ul FastAP™ SAP (Fermentas) and incubated at 37°C for 20 min followed by thermal inactivation at 85°C for 10 minutes. 2.5 μl of mastermix (1 μl 5× T7 R&DNA buffer, 0.25 μl 10 mM NTP/dNTP mix, 0.5 μl DTT, 0.25 T7 R&DNA Polymerase (Epicentre Biotechnologies) and 0.5 μl RNAse-Free water) was added to 2.5 μl PCR/SAP mix and incubated for 2 hours at 37°C l. Afterwards, 1 μl of RNAse A was added to each sample followed by incubation for 1 hour at 37°C. The sample was diluted to 15 μl with RNAse free water and 2 mg Dowex Cation Exchange Resin was added to each well. In all, four separate cleaved samples were generated from each DNA sample, two using a T7 promoter fused to a forward primer and two from a T7 promoter fused to a reverse primer. The matrix used was HPA (3-Hydroxypicolonic acid) with the concentration 35 mg/ml in 50% water and acetonitrile (v/v). Diammoniumcitrate (7.5 mg/ml) was used as co-matrix. 0.5 μl of the matrix solution was added to a Bruker™ Polished Steel 384-well plate and allowed to dry completely before addition of 0.5 μl sample on top of the dried HPA droplet. The finished sample was then analyzed in the positive ion mode using 400 ns delayed ion extraction. The laser was operated at a few percent over threshold values. The generated spectra were compared to the predicted mass-peaks of the sequence and several wild type spectra. Samples showing a marked deviance from wt were sequenced by Eurofins-MWG (Ebersberg, Germany).

## Authors' contributions

AC optimised and performed the EMS mutagenesis and was responsible for the logistics of the whole operation. AC, PS, MB, MN, TC and OO participated out-sowing, seed collection and harvesting of the M1 and M2 populations and helped with the DNA preparations. AC cloned *AsPAL1 *and identified the low lignin mutants and VV made the lignin quantifications. PS cloned *AsCslF6 *and conducted the RAPD experiments. PS and ML optimised the MALDI-TOF protocol for TILLING and identified the mutations in *AsPAL1 *and *AsCslF6 *genes. OO planned and supervised the work and wrote the manuscript together with AC and PS. All authors read and approved the final manuscript.
